# Obituary

**Published:** 1905-06-15

**Authors:** 


### OBITUARY.

Dr. Clarke Lamotte Goddard, of San Francisco, Cal., died at his home March 30th, 1905, at the age of fifty-six. He was born in Beloit, Wis., and graduated from Beloit College with the A. B. degree in 1872, and took up his dental degree in the Philadelphia Dental College in 1874. He began practice in San Francisco in March, 1875, and practiced there for just thirty years. He had a large and lucrative practice. He was for many years a teacher in the University of California dental department. He was a writer and student and was well known throughout the country. He was a courteous gentleman and highly esteemed by all who came to know him. His work for the profession was invaluable and he will be sadly mourned by a large number of professional men all through the country. He leaves a wife and two children.

